# Factors associated with diabetes knowledge, attitudes and practices among people aged 18 and over in the commune of Niakhene in Senegal

**DOI:** 10.1371/journal.pgph.0002265

**Published:** 2024-03-07

**Authors:** Amadou Ibra Diallo, Cheikh Mbacke Dieng, Jean Augustin Diegane Tine, Oumar Bassoum, Fatoumata Binetou Diongue, Mouhamadou Faly Ba, Ibrahima Ndiaye, Mbayang Ndiaye, Adama Faye, Ibrahima Seck

**Affiliations:** 1 Department of Preventive Medicine and Public Health Faculty of Medicine, Pharmacy and Odontology (FMPO), Cheikh Anta Diop University (UCAD), Dakar, Senegal; 2 Health and Development Institute (ISED), Cheikh Anta Diop University (UCAD), Dakar, Senegal; 3 Faculty of Medicine, Pharmacy and Odontology, Cheikh Anta Diop University (UCAD), Dakar, Senegal; Universiti Malaya, MALAYSIA

## Abstract

More than 422 million people worldwide have diabetes in 2016, and 1.6 million deaths are attributed to diabetes each year. Knowledge of preventive measures would enable the adjustment of preventive policies. Hence this study on knowledge and practices in rural Senegal. This was a cross-sectional, descriptive and analytical survey of subjects aged at least 18 and living in the commune of Niakhene, carried out in October 2020. A systematic random sample, stratified by sex and age group, was used. The questionnaire was based on the STEPS 2015 tool and a review of the literature. In addition to personal characteristics, the questionnaire was used to measure knowledge of symptoms, complications, risk factors, attitude to the disease and screening practices. Descriptive and analytical analyses were performed using R 4.0.2 software. A total of 300 subjects were surveyed. The average age was 35.3 years (+/-16.9), and 52.3% were women. Knowledge (62.7%) was associated with higher education (ORaj2.46{1.16–3.44}), awareness by healthcare staff (ORaj2.88{1.60–5.34}), and a family history of diabetes (ORaj3.09{1.06–11.3}). The positive attitude (53%) was associated with male sex (ORaj1.98{2.07–7.52}), awareness via audio-visual information sources (ORaj3.87{2.07–7.52}), community awareness (ORaj 3.87{2.07–7.52}), existence of a family history of hypertension and knowledge of diabetes (ORaj3.34{2.5–7.69}). Screening was carried out in 34.3% of patients. The associated risk factors were male sex (ORaj 1.95{1.12–3.34}), higher education (ORaj2.49{1.12–559}) and positive attitudes to diabetes (ORaj1.83{1.04–3.26}). One of the most effective interventions against this disease is the adoption of preventive measures which involve early detection and strengthening communication for more effective prevention.

## Introduction

Diabetes is now a global public health problem. According to World Health Organization (WHO) estimates, diabetes affected more than 422 million people in 2016, with 1.6 million deaths [1]. A more recent estimate suggests that the number of cases worldwide will reach 693 million people in 2045 [2].

In sub-Saharan Africa, more than 12 million people suffered from diabetes in 2014. The number of diabetes-related deaths was estimated at 330,000 in the same year. Furthermore, it should be noted that less than 1% of healthcare expenditure is allocated to this burden [1].

In Senegal, the 2015 STEPS survey found a national prevalence rate of 2.1% [3]. In 2016, the proportional mortality rate for diabetes in the general population was 3% [4].

Knowledge about diabetes could encourage people to adopt preventive measures and promote good attitudes and practices [5, 6].

Studies have shown that knowledge of the disease can be influenced by level of education [7–9], family history of diabetes [8–10], personal characteristics such as age, sex, socio-economic status, marital status and exposure to health education [11, 12]. One of the most effective interventions against the disease is the adoption of preventive measures through early detection. However, this practice is not yet systematic among populations in developing countries. In Africa, it is estimated that two-thirds of at-risk populations are either undiagnosed or unaware that they have diabetes [13].

However, knowledge of preventive measures or early detection of diabetes can help minimize the potentially disabling complications of vascular and neurological damage, with strokes having a major impact on quality of life, and even potentially fatal sudden death secondary to acute ischemic heart disease [13]. Raising awareness could also help reduce socio-cultural misconceptions about existence, diagnosis and treatment of diabetes. In the context of poor-income countries, awareness-raising could also limit recourse to traditional medicine, which is a source of delayed consultation while increasing acute complications such as diabetic coma and premature death [5, 6].

In Senegal, the national STEPS survey (2015) on WHO recommendation showed that glycemic status was ignored by 84.7% of the population [3]. Similarly, in Sri Lanka in 2017, this proportion represented more than half the participants in the study by Gall et al. [7]. The latter also showed that screening was associated with socio-economic status. People with a good socio-economic status had more regular blood glucose testing than people with a low socio-economic status [7].

In Senegal, most studies have focused on the prevalence of diabetes in the population, both in urban areas [14], rural [13] or at national level with the STEPS survey.

However, few studies have assessed knowledge. Hence this study of the factors associated with knowledge, attitudes and practice of diabetes screening in the commune of Niakhene in central Senegal. It will enable the adjustment of preventive policies and strategies for a more effective response to diabetes, under the coordination of the Non-Communicable Disease Control Division of Senegal’s Ministry of Health and Social Action, through the availability of evidence-based data aimed at promoting prevention, early detection and management of diabetes in Senegal.

### Study framework

The rural commune of Niakhène is part of the Meckhe health district which was created in 1983 following the sectoral division of the geographical area of the Tivaouane medical district. The Meckhe district is located in the department of Tivaouane in the Thies region of Senegal. Its surface area is 1516 km^2^ with a population of 181211 inhabitants in 2018 (ANSD), i.e. 119 inhabitants per km^2^. The district is exclusively Muslim, although traditional animist beliefs and practices remain. Families are large and live in concessions with several households. The vast majority of households are poor and their main economic activities are agriculture, livestock breeding, handicrafts and petty trade. Agriculture is dominated by the cultivation of millet, groundnuts and cassava. Market gardening is also important, with onions, cabbage and tomatoes being the main crops.

In terms of health, the district has 21 health posts, including 18 in rural areas, a type 1 referral health center in the commune of Meckhe and 72 health huts. The commune of Niakhene has 2 health posts and an isolated maternity unit.

There are no private or semi-public health facilities. The target population located less than 5 km from a health post is estimated at 60% of the total population.

## Method

### Type and population of study

This was a cross-sectional, descriptive and analytical survey of people aged at least 18 at the time of the survey, living in the commune of Niakhene for at least six months. The study was carried out during the second half of October 2020.

### Sampling

The study included all Senegalese nationals aged 18 and over, who stayed in the study area for at least 6 months.

In order to have significant results to describe attitude and practical knowledge with satisfactory power, the number of study participants was calculated using the Schwartz formula N = ε^2^ PQ / i^2^, taking into account the following parameters:

- Standard error (for an alpha error risk of 5%) = 1.96
- Expected prevalence P = 5.9% (Prevalence of diabetes at national level found in the 2015 STEPS survey in Senegal among people aged over 60) [3].
- Q is the complement of P, so Q = 1-P
- Accuracy (i) = 5%.

These parameters gave a necessary number of subjects of 85 people. For greater power, the size was increased to 300 individuals.

Sampling was based on a stratified systematic random sample [15] in clusters of 10 individuals, i.e. 30 clusters distributed within the villages of the commune of Niakhene.

Using the population database for the commune of Niakhène obtained from the last population census, we listed all 105 the villages in the commune. We then sorted the villages according to population size, from the smallest to the largest, and divided the total population of the commune by the number of 30 clusters. This gave us a sampling step and we took a random number between 1 and the sampling step, then added this step successively until we had 30 clusters from the total of 105 villages.

At each selected cluster level, a stratification proportional to the size of the population by age and sex was applied for greater representativeness. The proportions of the population by sex (male and female) and by age group (18–25, 25–40, 40–60 and 60+) were determined and then multiplied by the number of 10 people in the cluster. This gave the quota to be surveyed by age group and sex within the cluster. This was carried out for all 30 clusters (villages) in order to have all the available quotas.

Once in the cluster, the itinerary method was used to guide the interviewers to the concessions. After randomly selecting an intersection of several roads, the interviewer used his pen to choose a direction at random. All the concessions on this street/road were included until 10 people per cluster had been obtained, broken down by sex and age. A single person was selected at household level when several people met all the selection criteria by drawing lots. When the selected street failed to reach the target, the first street on the right was chosen until the target was reached.

### Data collection

Data were collected using a pre-coded questionnaire to answer the research questions, based on a literature search on NCDs by reference organizations such as the World Health Organization [16]. We included also articles in different countries evaluating diabetes knowledge, attitudes and practices [17, 18].

The finalized questionnaire was then saved on an electronic terminal using ODK Collect software (Open Data Kit ODK), which was synchronized with a server via the internet connection. This enabled the data to be recorded as the individual face-to-face interviews within the households were validated, with simultaneous transmission to a memory card and a secure server.

Each interviewer had a pre-established base of quotas according to gender and age group in each selected cluster (village) after application of the sampling procedures.

### Operational definition of variables

Average scores for knowledge and attitude to diabetes were calculated, and practice was determined on the basis of the question on whether screening had been carried out, using a dichotomous response method.

To calculate the average knowledge and attitude score, participants were asked to respond to a 5-point likert scale. The average scores were used to divide the participants into two groups: good and bad. For knowledge and attitude questions. Respondents with above-average scores were considered to have good knowledge and attitude, and below-average scores were considered to have poor knowledge and attitude. This procedure has been used in CAP studies on cardiovascular risk factors [18, 19].

### Data analysis

At the end of the survey, the data were extracted (S1 Data), compiled and cleaned before being analyzed using R 3.4.4 software.

Quantitative variables were described by mean with standard deviation, median with extremes, and qualitative variables by frequency.

For the analytical study, variables were cross-tabulated to reflect some issues raised in the objectives related to the relationships between personal characteristics and knowledge, attitudes and practices. The Chi-square test was used with an alpha risk of 5%. To account for confounding factors in the multivariate analysis, all variables with p values below 0.25 in the bivariate analysis were kept for the initial model [20, 21]. The top-down stepwise selection procedure was used to build the final model. Variables that did not improve the model were removed one by one. The likelihood ratio test was used to compare the nested models. The adequacy of the model was studied using the Hosmer Lemeshow [22].

### Ethics

The approval of the Research Ethics Committee (CER) of the Université Cheikh Anta Diop de Dakar was obtained before the start of the activities, bearing the reference number O25/2020/CER/UCAD.

Free, informed and signed consent was obtained from each interviewee prior to the interview. Interviewees could stop the interview at any time and even withdraw from the study without prejudice. Anonymity was respected and the results were kept confidential. The identity of the individuals who agreed to participate was not mentioned on the data collection tools or in the use of the results. Anonymity has been respected, and any identifying information was included in the database.

Any remuneration or compensation were given to study participants. The subjects covered remain general. The main benefit of this study will be a better knowledge of preventive measures and an improved diabetes response policy.

## Result

### Descriptive study

The study involved 300 individuals whose mean age was 35.3 (±16.7) years, with a median of 30 years and extremes ranging from 18 to 83 years. The most representative age group was 25–39, with 37.7% of respondents. The majority of respondents were married (65.7%) and uneducated (67.7%). Nearly 75% of the population were unemployed (40%) and predominantly belonged to the socio-economic middle quintile. The most common family history was hypertension (42.0%), followed by diabetes (9.0%) and stroke (5.7%) (Table 1).

**Table 1:** Breakdown by personal characteristics (n = 300).

Personal characteristics	Absolute frequency	Relative frequency
	(n)	(%)
**Age range of respondents**		
Under 25	98	*32*,*7*
Between 25 and 39	113	*37*,*7*
Between 40 and 59	48	*16*,*0*
Over 60s	41	*13*,*7*
**Gender**		
Female	157	*52*,*3*
Male	143	*47*,*7*
**Marital status**		
Married	197	*65*,*7*
Single	83	*27*,*7*
Widowed	15	*5*,*0*
Divorced	5	*1*,*7*
**Level of education**		
Without instruction	203	*67*,*7*
Primary	51	*17*,*0*
Secondary and above	46	*15*,*3*
**Profession**		
Shrew/unemployed	120	*40*,*0*
Farmer/breeder	67	*22*,*3*
Retailer	42	*14*,*0*
Student / Pupil	19	*6*,*3*
Worker	15	*5*,*0*
Senior executive	2	*0*,*7*
Other	35	*11*,*7*
**Socio-economic well-being**		
Poorer	46	*15*,*3*
Poor	46	*15*,*3*
Medium	75	*25*,*0*
Rich	69	*23*,*0*
Richer	64	*21*,*3*
Family history 1^er^ degree		
HTA	126	*42*,*0*
Diabetes	27	*9*,*0*
AVC	17	*5*,*7*

**Fig 1:**
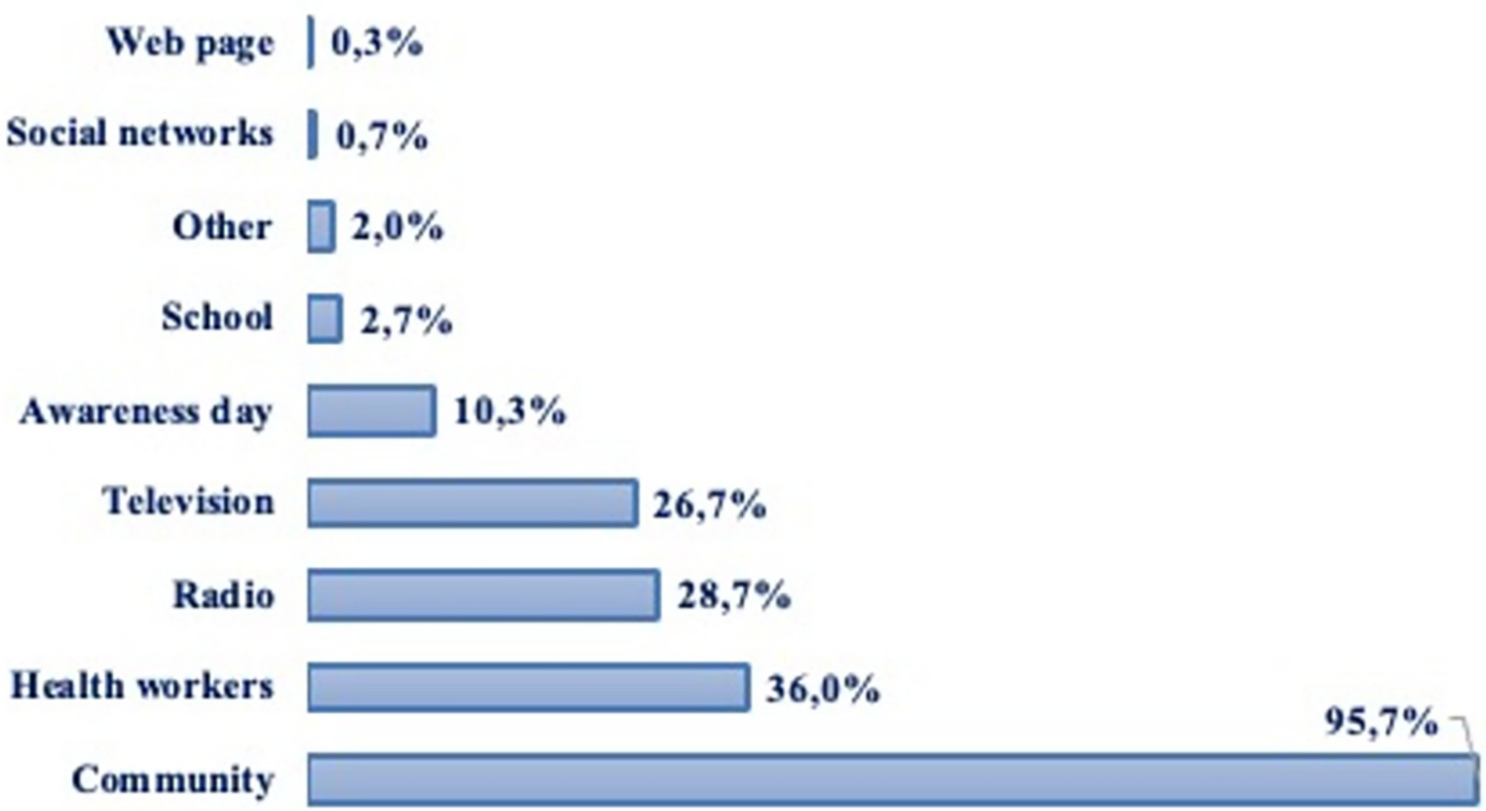
Distribution of sources of information on diabetes (n = 300).

The community (95.7%), healthcare staff (36.0%), television (28.7%) and radio (26.7%) were the main sources of information about diabetes in our study population. Social networks and web pages accounted for less than 1% of the communication channels cited (Fig 1).

A third (33%) of respondents strongly agreed that diabetes was a non-communicable disease, compared with 11.7% who disagreed. Diabetes had a genetic component according to 66.3% of respondents and did not only affect the elderly according to 67.6% of participants interviewed (Table 2).

**Table 2:** Distribution of the study population according to knowledge of diabetes (n = 300).

Knowledge of diabetes	Yes, absolutely 5		Yes, rather 4		Neutral in response 3		No, rather not 2		No, not at all 1		Don’t know 0	
	n	%	n	%	n	%	n	%	n	%	n	%
**General knowledge**												
Non-communicable disease	99	*33*,*0*	69	*23*,*0*	27	*9*,*0*	34	*11*,*3*	35	*11*,*7*	36	*12*,*0*
A disease with a permanent cure	74	*24*,*7*	37	*12*,*3*	23	*7*,*7*	99	*33*,*0*	49	*16*,*3*	18	*6*,*0*
Genetic component	121	*40*,*3*	78	*26*,*0*	31	*10*,*3*	17	*5*,*7*	10	*3*,*3*	43	*14*,*3*
Diabetes only affects the elderly	32	*10*,*7*	35	*11*,*7*	25	*8*,*3*	88	*29*,*3*	115	*38*,*3*	5	*1*,*7*
Diabetes is diagnosed by a blood test	147	*49*,*0*	106	*35*,*3*	23	*7*,*7*	2	*0*,*7*	2	*0*,*7*	20	*6*,*7*
**Knowledge of signs**												
Excessive hunger	96	*32*,*0*	93	*31*,*0*	37	*12*,*3*	26	*8*,*7*	12	*4*,*0*	36	*12*,*0*
Weight loss	144	*48*,*0*	105	*35*,*0*	13	*4*,*3*	10	*3*,*3*	6	*2*,*0*	22	*7*,*3*
Increased appetite	90	*30*,*0*	99	*33*,*0*	39	*13*,*0*	27	*9*,*0*	10	*3*,*3*	35	*11*,*7*
Increased frequency of urination	140	*46*,*7*	101	*33*,*7*	24	*8*,*0*	4	*1*,*3*	2	*0*,*7*	29	*9*,*7*
Repeated skin abscesses	61	*20*,*3*	86	*28*,*7*	45	*15*,*0*	21	*7*,*0*	12	*4*,*0*	75	*25*,*0*
**Knowledge of complications**												
Heart failure or heart attack	66	*22*,*0*	76	*25*,*3*	54	*18*,*0*	21	*7*,*0*	5	*1*,*7*	78	*26*,*0*
Renal insufficiency	54	*18*,*0*	77	*25*,*7*	60	*20*,*0*	14	*4*,*7*	1	*0*,*3*	94	*31*,*3*
Eye problems or blindness	95	*31*,*7*	86	*28*,*7*	44	*14*,*7*	10	*3*,*3*	2	*0*,*7*	63	*21*,*0*
Cerebral diseases Stroke	87	*29*,*0*	70	*23*,*3*	49	*16*,*3*	15	*5*,*0*	3	*1*,*0*	76	*25*,*3*
Amputation of a limb	178	*59*,*3*	82	*27*,*3*	16	*5*,*3*	5	*1*,*7*	0	*0*,*0*	19	*6*,*3*
**Knowledge of risk factors**												
Family history of diabetes	117	*39*,*0*	87	*29*,*0*	31	*10*,*3*	21	*7*,*0*	15	*5*,*0*	29	*9*,*7*
Being overweight and/or obese	106	*35*,*3*	117	*39*,*0*	23	*7*,*7*	14	*4*,*7*	10	*3*,*3*	30	*10*,*0*
Being physically inactive	119	*39*,*7*	117	*39*,*0*	25	*8*,*3*	14	*4*,*7*	4	*1*,*3*	21	*7*,*0*
Bad eating habits	154	*51*,*3*	107	*35*,*7*	13	*4*,*3*	3	*1*,*0*	2	*0*,*7*	21	*7*,*0*

The respondents (63%) agreed that hunger was one of the warning signs of diabetes. Weight loss was mentioned by 83% of respondents, increased appetite 63% and increased frequency of urination 70.4% (Table 2).

Assessment of the level of knowledge of diabetes complications revealed that 47.3% of respondents mentioned that diabetes could be responsible for heart damage, 43.7% mentioned kidney damage, 60.4% mentioned eye damage and 52.3% mentioned neurological damage. The most common complication was limb amputation, mentioned by 86.6% of respondents (Table 2).

More than half of respondents strongly agreed that poor dietary habits were a risk factor for diabetes (51.3%). And more than a third strongly agreed that a family history of diabetes (39.0%) and physical inactivity (39.7%) increased the risk of developing diabetes (Table 2).

The results showed that 35.4% of respondents did not find it embarrassing to talk to their loved ones about their diabetes while 91.4% of respondents were in favor of diabetes screening. The study also found that 90% of participants believed that reducing sugar consumption would protect against the onset of diabetes. The populations surveyed recommended physical activity (85.4%), maintaining a normal weight (75.4%) and controlling blood sugar levels (91.3%) as good ways of preventing diabetes (Table 3).

**Table 3:** Distribution of participants according to attitudes to diabetes (n = 300).

Attitudes towards diabetes	Yes, absolutely 5		Yes, rather 4		Neutral in response 3		No, rather not 2		No, not at all 1		Don’t know 0	
	n	%	n	%	n	%	n	%	n	%	n	%
If you were diabetic, would you mind if others knew?	65	21,7	41	13,7	29	9,7	61	20,3	101	33,7	3	1,0
Do you think you should be tested for diabetes?	188	62,7	92	30,7	12	4,0	4	1,3	3	1,0	1	0,3
Do you think family members should be screened for diabetes	186	62,0	91	30,3	14	4,7	5	1,7	2	0,7	2	0,7
Do you think you should limit your sugar intake?	163	54,3	107	35,7	7	2,3	20	6,7	1	0,3	2	0,7
Do you think diabetes seriously affects activities of daily living?	135	45,0	104	34,7	23	7,7	23	7,7	6	2,0	9	3,0
Do you think physical activity can prevent the risk of developing diabetes?	149	49,7	107	35,7	20	6,7	4	1,3	1	0,3	19	6,3
Do you think that maintaining a normal weight is important in preventing diabetes?	110	36,7	116	38,7	26	8,7	12	4,0	3	1,0	33	11,0
Complications of diabetes can be avoided if blood sugar levels are well controlled	153	51,0	113	37,7	11	3,7	2	0,7	0	0,0	21	7,0
Do you think that checking your blood sugar often helps prevent diabetes?	165	55,0	109	36,3	9	3,0	2	0,7	0	0,0	15	5,0

A total of 103 individuals, or 34.3% of the study population, had been screened for diabetes in the past. Of these, 31.6% had been screened during a routine consultation at a health facility and 1.3% during a mass screening campaign. Of the 103 people screened, 9 were diagnosed with diabetes. This represents a prevalence of 3% of old cases of diabetes (Table 4).

**Table 4:** Distribution of the study population according to past diabetes screening (N = 300).

Screening practice	Absolute frequency	Relative frequency
	(n)	(%)
**Screening for diabetes in the past**		
Yes	103	34,3
No	197	65,7
**Place of screening**		
General consultation (health post, health centre, hospital)	95	31,6
Free consultation days (mass campaign)	4	1,3
At the pharmacy	2	0,6
At home	1	0,3
Other	1	0,3
**Time since last screening**		
0 to 6 months	36	12,0
7 to 12 months	32	10,6
More than a year	35	11,6
**Positive screening result (diabetic)**	9	3,0

### Analytical study

#### Bivariate

Knowledge was assessed on a set of 19 items measured by a 5-point maximized Likert scale. The mean knowledge score was 64.7 (+/-18.2). Good knowledge, defined as a knowledge score above or equal to the mean, was found in 62.7% of the study population. Factors associated with knowledge were age, education, awareness by healthcare staff, and family history of hypertension and diabetes (Tables 5 and 6).

**Table 5:** Personal characteristics and knowledge, attitudes and practices in the bivariate model.

Variable	Good knowledge		P value	A good attitude		P value	Practical screening		P value
	Yes (%)	No (%)		Yes (%)	No (%)		Yes (%)	No (%)	
				N = 159	N = 141				
							N = 103	N = 197	
	N = 188	N = 112							
**Gender**									
Female	104 (66.2)	53 (33.8)	0.222	76 (48.4)	81 (51.6)	0.120	63 (40.1)	94 (59.9)	**0.036**
Male	84 (58.7)	59 (41.3)		83 (58.0)	60 (42.0)		40 (28.0)	103 (72.0)	
**Age range**									
[18–25 years [	59 (60.2)	39 (39.8)	**0.025**	53 (54.1)	45 (45.9)	0.273	21 (21.4)	77 (78.6)	**<0.001**
[25–40 years [	73 (64.6)	40 (35.4)		56 (49.6)	57 (50.4)		35 (31.0)	78 (69.0)	
[40–59 years old [	37 (77.1)	11 (22.9)		31 (64.6)	17 (35.4)		24 (50.0)	24 (50.0)	
60 and over	19 (46.3)	22 (53.7)		19 (46.3)	22 (53.7)		23 (56.1)	18 (43.9)	
**Level of education**									
Without instruction	116 (57.1)	87 (42.9)	**0.012**	95 (46.8)	108 (53.2)	**0.008**	68 (33.5)	135 (66.5)	0.535
Primary	36 (70.6)	15 (29.4)		34 (66.7)	17 (33.3)		16 (31.4)	35 (68.6)	
Secondary/Higher	36 (78.3)	10 (21.7)		30 (65.2)	16 (34.8)		19 (41.3)	27 (58.7)	
**Profession**									
Housewife/Unemployed	77 (64.2)	43 (35.8)	0.323	55 (45.8)	65 (54.2)	0.099	46 (38.3)	74 (61.7)	0.169
Other	29 (58.0)	21 (42.0)		33 (66.0)	17 (34.0)		14 (28.0)	36 (72.0)	
Senior executive	2 (100)	0 (0.00)		0 (0.00)	2 (100)		2 (100)	0 (0.0)	
Retailer	23 (54.8)	19 (45.2)		21 (50.0)	21 (50.0)		14 (33.3)	28 (66.7)	
Cultivator	38 (60.3)	25 (39.7)		32 (50.8)	31 (49.2)		18 (28.6)	45 (71.4)	
Breeder	4 (100)	0 (0.00)		4 (100)	0 (0.00)		3 (75.0)	1 (25.0)	
Student / Pupil	15 (78.9)	4 (21.1)		14 (73.7)	5 (26.3)		6 (31.6)	13 (68.4)	
**Quintile**									
Poorer	26 (56.5)	20 (43.5)	0.429	22 (47.8)	24 (52.2)	0.172	16 (34.8)	30 (65.2)	0.122
Poor	28 (60.9)	18 (39.1)		20 (43.5)	26 (56.5)		10 (21.7)	36 (78.3)	
Medium	48 (64.0)	27 (36.0)		36 (48.0)	39 (52.0)		22 (29.3)	53 (70.7)	
Rich	40 (58.0)	29 (42.0)		41 (59.4)	28 (40.6)		30 (43.5)	39 (56.5)	
Richer	46 (71.9)	18 (28.1)		40 (62.5)	24 (37.5)		25 (39.1)	39 (60.9)	

**Table 6:** Family history, sources of information and knowledge, attitudes and practices in the bivariate model.

Variable	Good knowledge			P value	A good attitude		P value	Practical screening		P value
	Yes (%)		No (%)		Yes (%)	No (%)		Yes (%)	No (%)	
**Information about diabetes on television**										
No	136 (61.8)		84 (38.2)	0.712	100 (45.5)	120 (54.5)	**<0.001**	77 (35.0)	143 (65.0)	0.790
Yes	52 (65.0)		28 (35.0)		59 (73.8)	21 (26.2)		26 (32.5)	54 (67.5)	
**Diabetes information on the radio**										
No	135 (63.1)		79 (36.9)	0.917	100 (46.7)	114 (53.3)	**0.001**	71 (33.2)	143 (66.8)	0.596
Yes	53 (61.6)		33 (38.4)		59 (68.6)	27 (31.4)		32 (37.2)	54 (62.8)	
**Information on diabetes at awareness days**										
No	170 (63.2)		99 (36.8)	0.716	135 (50.2)	134 (49.8)	**0.007**	86 (32.0)	183 (68.0)	**0.019**
Yes	18 (58.1)		13 (41.9)		24 (77.4)	7 (22.6)		17 (54.8)	14 (45.2)	
**Information on diabetes from healthcare staff**										
No	106 (55.2)		86 (44.8)	**0.001**	97 (50.5)	95 (49.5)	0.305	60 (31.2)	132 (68.8)	0.170
Yes	82 (75.9)		26 (24.1)		62 (57.4)	46 (42.6)		43 (39.8)	65 (60.2)	
**Information about diabetes from friends and the community**										
No	10 (76.9)		3 (23.1)	0.384	6 (46.2)	7 (53.8)	0.825	4 (30.8)	9 (69.2)	0.099
Yes	178 (62.0)		109 (38.0)		153 (53.3)	134 (46.7)		99 (34.5)	188 (65.5)	
**Family history of hypertension**										
No	98 (56.3)		76 (43.7)	**0.011**	81 (64.3)	45 (35.7)	**0.001**	48 (27.6)	126 (72.4)	**0.006**
Yes	90 (71.4)		36 (28.6)		78 (44.8)	96 (55.2)		55 (43.7)	71 (56.3)	
**Family history of diabetes**										
No	165 (60.4)		108 (39.6)	**0.020**	140 (51.3)	133 (48.7)	0.090	88 (32.2)	185 (67.8)	**0.026**
Yes	23 (85.2)		4 (14.8)		19 (70.4)	8 (29.6)		15 (55.6)	12 (44.4)	
**Family history of stroke**										
No	175 (61.8)		108 (38.2)	0.340	146 (51.6)	137 (48.4)	0.081	94 (33.2)	189 (66.8)	0.161
Yes	13 (76.5)		4 (23.5)		13 (76.5)	4 (23.5)		9 (52.9)	8 (47.1)	
**Knowledge about diabetes**										
Wrong	-		-	-	37 (33.0)	75 (67.0)	**<0.001**	32 (28.6)	80 (71.4)	0.106
Good	-		-	-	122 (64.9)	66 (35.1)		71 (37.8)	117 (62.2)	
**Attitudes towards diabetes**										
Wrong	-		-	-	-	-	-	36 (25.5)	105 (74.5)	**0.004**
Good	-		-	-	-	-	-	67 (42.1)	92 (57.9)	

The attitude score was determined from 9 items assessed by a 5-point maximized Likert scale. The mean attitude score was 36.4 (+/-5.9). A positive attitude was defined as a score above or equal to the mean, and was found in 53% of the study population. Risk factors associated with attitudes to diabetes included education, awareness through audio-visual information sources, community awareness, family history of hypertension and knowledge of diabetes (Tables 5 and 6).

In this study, past practice of screening was found in 34.3% of the respondents. Risk factors associated with past screening were gender, age, community awareness, family history of hypertension and diabetes, and attitudes towards diabetes. These factors were identified both in those with no history and in the sample as a whole (Tables 5 and 6).

#### Multivariate

In terms of knowledge, people with at least secondary education had 2.46 (OR aj [1.08–6.00]) times more knowledge about diabetes than those uneducated. Those who received information about diabetes from healthcare staff also had almost 2.8 times (OR aj [1.60–5.34]) more knowledge, while those with a family history of diabetes had 3 (OR aj [1.06–11.3]) times more knowledge (Table 7).

**Table 7:** Final multivariate models of factors associated with knowledge, attitudes and practices.

Variable	Good knowledge			P value	A good attitude		P value	Practical screening		P value
	ORaj		95% CI		ORaj	95% CI		ORaj	95% CI	
**Gender**										
Male	Ref		Ref	Ref	Ref	Ref	Ref	Ref	Ref	Ref
Female	1.38		0.83–2.31	0.200	**0.5**	**0.29–0.86**	0.013*	**1.95**	**1.12–3.44**	0.019*
**Age range**										
[18–25 years [	Ref		Ref	Ref	-	-	-	Ref	Ref	Ref
[25–40 years [	1.11		0.59–2.09	0.800	-	-	-	1.80	0.89–3.71	0.100
[40–59 years old [	2.12		0.90–5.22	0.091	-	-	-	**5.17**	**2.20–12.6**	<0.001*
60 and over	0.63		0.26–1.50	0.300	-	-	-	**10.8**	**4.29–28.7**	<0.001*
**Level of education**										
Without instruction	Ref		Ref	Ref	Ref	Ref	Ref	Ref	Ref	Ref
Primary	1.75		0.86–3.68	0.13	1.59	0.77–3.35	0.2	1.18	0.54–2.55	0.7
Secondary/Higher	**2.46**		**1.08–6.00**	0.038*	1.14	0.53–2.45	0.7	**2.49**	**1.12–5.59**	0.025*
**Information about diabetes on television**										
No	-		-	-	Ref	Ref	Ref	-	-	-
Yes	-		-	-	**3.87**	**2.07–7.52**	<0.001*	-	-	-
**Information on diabetes at awareness days**										
No	Ref		Ref	Ref	Ref	Ref	Ref	Ref	Ref	Ref
Yes	0.59		0.26–1.38	0.200	**5.59**	**2.20–15.9**	<0.001*	2.15	0.93–5.06	0.074
**Information on diabetes from healthcare staff**										
No	Ref		Ref	Ref	-	-	-	-	-	-
Yes	**2.88**		**1.60–5.34**	<0.001*	-	-	-	-	-	-
**Family history of hypertension**										
No	Ref		Ref	Ref	Ref	Ref	Ref	Ref	Ref	Ref
Yes	1.63		0.94–2.85	0.082	**2.43**	**1.41–4.25**	0.002*	**2.75**	**1.53–5.05**	<0.001*
**Family history of diabetes**										
No	Ref		Ref	Ref	-	-	-	-	-	-
Yes	**3.09**		**1.06–11.3**	0.046*	-	-	-	-	-	-
**Knowledge about diabetes**										
Wrong	-		-	-	Ref	Ref	Ref	-	-	-
Good	-		-	-	**3.34**	**2.50–7.69**	<0.001*	-	-	-
**Attitudes towards diabetes**										
Wrong	-		-	-	-	-	-	Ref	Ref	Ref
Good	-		-	-	-	-	-	**1.83**	**1.04–3.26**	0.037*

**ORaj:** Adjusted Odd Ratio; **CI:** Confidence Interval; *: Significance

Males had a more positive attitude towards diabetes (OR aj = 1.98 [1.16–3.44]). Sources of information about diabetes via television (OR aj = 3.87 [2.07–7.52]) and awareness days (OR aj = 5.59 [2.20–15.9]) were factors associated with the development of positive attitudes towards the disease. Positive attitudes were also higher among those with a family history of hypertension (OR aj = 2.43 [1.41–4.25]) and those with a good knowledge of diabetes (OR aj = 3.34 [2.50–7.69]) (Table 7).

Men (OR aj = 1.95 [1.12–3.44]), those with a high level of education (OR aj = 2.49 [1.12–5.59]) and those with a family history of hypertension (OR aj = 2.75 [1.53–5.05]) were more likely to be screened for diabetes. The practice of screening was also associated with age, better in the 40–59 age group (OR aj = 5.17 [2.20–12.6]) and the 60+ age group (OR aj = 10.8 [4.29–28.7]) than in the younger 18–25 age group. Those with a positive attitude towards diabetes (OR aj = 1.83 [1.04–3.26]) were more likely to undergo screening (Table 7).

## Discussion

This study of diabetes knowledge, attitudes and practices was carried out in the commune of Niakhene, Senegal, among 300 adults aged 18 to 83.

As regards knowledge of diabetes, a third of respondents said that it was a non-communicable disease and 33% were sure it was a disease that could not be permanently cured. This finding on chronicity is lower than the results of the 2015 study by Debre Tabor et al, where 51.3% of respondents said diabetes is incurable [23]. Good knowledge of the signs of diabetes, average knowledge of complications and low knowledge of risk factors were in line with the results of a study carried out in a semi-urban area of Oman in 2008, which showed that respondents had a good understanding of the definition (46.5%), symptoms (57%) and complications of diabetes (55.1%) [24]. Another study carried out in Ethiopia in 2017 found lower knowledge of the symptoms, risk factors (48%) and complications of diabetes (51.5%) [18]. Community awareness, healthcare staff and audiovisual media were the main sources of information about diabetes. This finding of the predominance of the source of information linked to the experience of illness in the community should prompt further reflection on communication strategies on cardiovascular risk factors in general and diabetes in particular by health authorities. To avoid misinformation, the health system should review diabetes awareness strategies by building the interpersonal and mass communication skills of healthcare workers and community actors in order to carry out community visits and multiply contact with the population at the healthcare facilities to emphasize the importance of early detection in preventing diabetes-related complications through screening at the time of consultations.

As part of primary prevention, the department in charge of combating non-communicable diseases in Senegal has reiterated its commitment in its 2017–2020 strategic plan to improve knowledge about CVDs by focusing on communication [25].

Overall knowledge based on the mean score of a 5-point Likert scale of 19 items was 62.7%. This level of overall knowledge of diabetes could be acceptable compared with the 47.4% of adequate knowledge found in a 2018 study in Pakistan [9]. Factors associated with knowledge of diabetes showed that educated people with at least secondary education were more likely to have better knowledge of diabetes. This could be explained by the fact that educated people are more attentive to communication and awareness issues regarding diabetes messages and do not hesitate to go in search of information [26]. Individuals who received diabetes education from healthcare personnel were more knowledgeable. Other studies have shown that, irrespective of the source of information on diabetes, people who have been reached by information messages have a better knowledge of the disease [27]. Experience of the disease and first-degree family antecedents, which are major cardiovascular risk factors, were also a predisposing factor for good knowledge of the disease. This was shown in the study by Kassahun et al [18] in Ethiopia in 2016 and Alsous et al in Jordan in 2019 [28]. This is because people with a family history of chronic disease, may develop a delicate sense of vulnerability that could increase their level of knowledge of the disease [29].

In addition, patients with diabetes who are receiving good therapeutic education can be play a key role in the prevention if they get involved in the fight against diabetes by raising awareness among their parents and relatives about preventive measures and screening.

The results showed that 35.4% of respondents did not find it embarassing to talk about their diabetes to friends and family, and that 91.4% of respondents were in favor of diabetes screening. Overall, 53.0% of the study population had a positive attitude towards diabetes. This proportion is in line with those noted in two Ethiopian studies in the commune of Bale (55.9%) in 2016 [18] and in the commune of Gandar in 2021 [19]. This is due to the expansion of healthcare services in Senegal, as set out in the National Health and Social Development Plan 2019–2028, which prioritizes the fight against non-communicable diseases through primary prevention programs [30]. The other reason could be the similarity of the study design used in these different studies. On the other hand, it is higher compared with studies conducted in rural settings in India in 2014 [31], Kenya in 2015 [32] and Saudi Arabia in 2018 [33] which found respectively 17.6%, 41.4% and 48% of good attitudes to diabetes.

Factors associated with positive attitudes included gender, access to information on diabetes via traditional sources, in this case television and awareness days, family history of hypertension and good knowledge of the disease. These results are consistent with the literature on the determinants of positive attitudes identified in studies conducted in Ethiopia [34] Jordan [28], South Africa [35]. Well-informed people have the opportunity to interact with others and share ideas and feelings. Clearly, as knowledge about diabetes increases, individuals will tend to participate in health education campaigns, watch television or listen to the radio, which is an effective way of obtaining safe and practical information about diabetes [19].

Nearly a third (34.3%) of the sample had undergone at least one diabetes screening. This proportion of blood glucose screening is close to the 32% reported in the Alqahtami study in Saudi Arabia in 2020 [36]. This may be a warning that raising awareness of diabetes screening is crucial. As the disease progresses, complications and socio-economic burdens multiply. Early detection of the disease certainly has an impact on quality of life, but above all it helps avoid complications and is a cost-effective disease prevention measure [16]. The practice of screening was significantly associated with gender, age, awareness, family history of hypertension and attitude. As individuals age, they may become more aware or concerned about their health, which may lead them to perform more tests in general, including blood glucose tests [37]. In Senegal, as in most sub-Saharan countries, a large proportion of health contributions are allocated to women’s health needs. This is because this is the age of reproductive, and women necessarily have a greater need for health services at this age [38]. The higher frequency of screening in people with a family history of cancer [28] is explained by the fact that people with a family history of chronic disease are more likely to develop the disease. This may increase their positive attitude towards the disease [29]. This was reflected in their practices, as they were more likely to undergo screening tests. The present study also found significant positive associations between attitude and level of practice, i.e., having a good attitude towards diabetes predicted diabetes screening. The study indicated that people with a positive attitude towards diabetes will willingly implement diabetes risk reduction activities [18].

### Limits

The cross-sectional nature of the study makes it difficult to establish a causal relationship between predictive factors and knowledge, attitudes and practices. It may also be a source of memory bias regarding past screening practice, as some people may have difficulty recalling certain information accurately. As this study was carried out in rural areas, this may constitute a bias in inferring results for rural areas. Nevertheless, the systematic drawing and quota approach constitute a method of guaranteeing the representativeness and, beyond that, the validity of the results in rural areas, pending estimates of national knowledge, attitudes and practices on non-communicable diseases in particular diabetes. The evaluation of these estimates could be integrated into the STEPS survey on diabetes prevalence, organized every 5 years, in order to better adapt prevention measures.

## Conclusion

Knowledge of diabetes was influenced by level of education, family history of diabetes and exposure to health education. One of the most effective interventions in the fight against this disease is the adoption of preventive measures through early detection. However, this practice is not yet systematic in some populations. Hence the need for healthcare staff to step up communication to improve attitudes to the disease, to enable more effective prevention by reducing the number of people who are unaware of their blood sugar levels, thereby reducing complications and premature deaths linked to diabetes.

## Supporting information

S1 Data Database. (XLSX)
